# B3GNT5 is a novel marker correlated with stem‐like phenotype and poor clinical outcome in human gliomas 

**DOI:** 10.1111/cns.13439

**Published:** 2020-07-16

**Authors:** Hang Yeon Jeong, Seo‐Young Park, Hyun‐Jin Kim, Seungju Moon, Seongsoo Lee, Seung Ho Lee, Sung‐Hak Kim

**Affiliations:** ^1^ Department of Animal Science College of Agriculture and Life Sciences Chonnam National University Gwangju Korea; ^2^ Gwangju Center Korea Basic Science Institute Gwangju Korea; ^3^ Department of Nano‐Bioengineering Incheon National University Incheon Korea

**Keywords:** B3GNT5, brain cancer, glioblastoma multiform, glioma stem cell

## Abstract

**Aims:**

Glioblastoma multiforme **(**GBM) is the most lethal tumor with a median patient survival of 14 to 15 months. Glioma stem cells (GSCs) play a critical role in tumor initiation and therapeutic resistance in GBM. B3GNT5 has been suggested as the key glycosyltransferase in the biosynthesis of the (neo‐) lacto series of glycosphingolipid. In this study, we evaluated the *B3GNT5* expression in GSCs as well as the correlation with clinical data in GBM.

**Methods:**

The mRNA levels of *B3GNT5* in normal astrocytes, four glioma cell lines, and four GSCs were evaluated using real‐time PCR. Small interference RNAs (siRNAs) were used to inhibit *B3GNT5* expression and analyze its ability to form neurospheres. Statistical analyses were conducted to determine the association with *B3GNT5* expression and tumor grade and GBM subtypes as well as patient survival using public datasets.

**Results:**

*B3GNT5* expression was significantly elevated in GSCs compared with normal astrocytes, glioma cell lines, and their matched differentiated tumor cells. Knockdown of *B3GNT5* in GSCs decreased the neurosphere formation. Patients with high *B3GNT5* expression had a short overall survival. B3GNT5 is correlated with classical and mesenchymal GBM subtypes.

**Conclusion:**

The findings suggest the central role of B3GNT5 in regulating malignancy of GBM.

## Graphical abstract

Expression of *B3GNT5*, the key glycosyltransferase in the biosynthesis of the glycosphingolipid, was significantly elevated in GSCs compared with normal astrocytes, glioma cell lines, and their matched differentiated tumor cells. Knockdown of *B3GNT5* in GSCs decreased the neurosphere formation. Patients with high *B3GNT5* expression had a short overall survival.

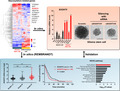

## 1 INTRODUCTION

Glioblastoma (GBM) is a grade IV brain tumor, which is one of the most malignant tumors associated with a reduced long‐term survival despite aggressive treatment strategies, such as surgery, radiotherapy, and chemotherapy.^1^ Recent studies have shown that glioma stem cells (GSCs), which resemble normal stem cells in terms of potential self‐renewal and terminal differentiation, are involved in tumor development, therapeutic resistance, and recurrence.^2^ Therefore, targeting the stem cell characteristics of GSCs is a novel breakthrough in GBM treatment.

Glycosphingolipids (GSLs) represent a large family of glycoconjugates found abundantly in cellular membranes. GSLs are classified into different series defined by their respective core structures. The major GSL types include ganglio‐, lacto‐, globo‐, isoglobo‐, and muco‐series.^3^ GSL series play a crucial role in cell adhesion, cell migration, regulation of signaling proteins, and binding of pathogens and toxins.^4^, ^5^

*B3GNT5* (UDP‐GlcNAc:β‐Gal β‐1,3‐*N*‐acetylglucosaminyltransferase 5) is a glycosyltransferase that transfers an *N*‐acetylglucosamine (GlcNAc) from uridine diphosphate (UDP)‐GlcNAc to galactose at the nonreducing end of carbohydrate chain. B3GNT5 is a key glycosyltransferase that mediates the biosynthesis of lacto‐ and neolactoseries of GSLs. B3GNT5 transfers *N*‐acetylglucosamine to the C‐3 position of galactose in lactosylceramide resulting in the synthesis of lactotriaosylceramide, and thus, B3GNT5 is also known as lactosylceramide synthase. Several studies reported the biological significance of B3GNT5‐mediated synthesis of glycolipids in B‐cell activation,^6^ pre‐implantation development,^7^ and development of nerve system,^8^, ^9^ The increased levels of B3GNT5 appear to be strongly correlated with the progression of breast cancer,^10^ lung cancer,^11^ and ovarian cancer.^12^ In addition, B3GNT5‐mediated glycosphingolipids play a key role in the differentiation of acute myeloid leukemia (AML) cells.^13^

However, the role of B3GNT5 in GSC differentiation remain is incompletely understood. Therefore, in this study, we investigated the association between the expression of B3GNT5 and the malignancy of GBM using the Repository for Molecular Brain Neoplasia Data (REMBRANDT), Gene Expression Omnibus (GEO) (GSE4536), and Ivy Glioblastoma Atlas Project. In addition, we further evaluated the effects of B3GNT5 on the maintenance of stemness in cancer stem cells (CSCs) using GSCs derived from primary human GBM tissues.

## 2 MATERIALS AND METHODS

### 2.1 Cell culture and reagents

Normal human astrocytes (NHA) were maintained in astrocyte media (Welgene) supplemented with 10% fetal bovine serum (FBS; HyClone, Thermo Fisher Scientific), and 1% penicillin‐streptomycin (Welgene). Glioma cells (A172, A1207, and U87MG) were cultured in Dulbecco's modified Eagle's medium (DMEM)/F12 (Welgene) supplemented with 10% fetal bovine serum (FBS; HyClone, Thermo Fisher Scientific) and 1% penicillin‐streptomycin (Welgene). GBM patient‐derived glioma stem cells (GSC11, GSC20, GSC23, and GSC267) were provided by The University of Texas MD Anderson Cancer Center. GSCs were cultured under stem cell culture conditions (NBE, serum‐free neurobasal media supplemented with growth factors). The NBE comprised DMEM/F12 (Welgene) supplemented with B27 (Gibco), 1% penicillin/streptomycin (Welgene), epidermal growth factor (EGF; 20 ng/mL; R&D Systems), and basic fibroblast growth factor (bFGF; 20 ng/mL; R&D Systems).^14^ Growth factors (bFGF and EGF) were added twice a week. To induce differentiation, GSC11 and GSC23 cells were cultured for 10 days in DMEM/F12 containing 10% FBS.

### 2.2 Quantitative reverse transcription‐PCR (RT‐qPCR)

Total RNA was extracted using RiboEx reagent (GeneAll) and purified using Hybrid‐R kit (GeneAll), according to the manufacturer's instructions. One microgram of mRNA was used to synthesize complementary DNA (cDNA) with Thermo Scientific RevertAid First‐Strand cDNA Synthesis Kit (Thermo Fisher Scientific). The RT‐qPCR was conducted on a CFX96 real‐time PCR detection system (Bio‐Rad Laboratories) using SYBR Premix Ex Taq (Takara Bio). The results of RT‐qPCR were evaluated as cycle threshold (Ct) values and, in turn, were quantified using the 2^−ΔΔCt^ method.^15^ Primer sequences used for RT‐qPCR amplification were as follows (5′ to 3′): 18S (loading control): forward (F), CAGCCACCCGAGATTGAGCA and reverse (R), TAGTAGCGACGGGCGGTGTG; *B3GNT5*: F, GGGCCTCGCTACCAATACTTG and R, CGGAACGTCGATCATAGTTTTCA; *CD15*: F, TTGGGACCTCCTAGTTCCAC and R, TGTAAGGAAGCCACATTGGA; *CD133*: F, CAGGTAAGAACCCGGATCAA and R, TCAGATCTGTGAACGCCTTG; *GFAP*: F, GGAACATCGTGGTGAAGACC and R, AGAGGCGGAGCAACTATCCT; *TUBB3*: F, AGTGTGAAAACTGCGACTGC and R, ACGACGCTGAAGGTGTTCAT; *S100β*: F, TCAAAGAGCAGGAGGTTGTG and R, TCGTGGCAGGCAGTAGTAAC.

### 2.3 Dataset preparation for B3GNT5 expression and survival analysis

Repository for Molecular Brain Neoplasia Data (REMBRANDT), Gene Expression Omnibus (GEO) (GSE4536), and Ivy Glioblastoma Atlas Project datasets were used for the analysis of *B3GNT5* expression. Data from REMBRANDT were obtained from GlioVis (https://gliovis.bioinfo.cnio.es/).

### 2.4 Transfection with small interfering RNA

Small interfering RNAs (siRNA) targeting the *B3GNT5* transcript were purchased from Bioneer (Daejeon). Sequences were as follows (5′ to 3′): si*B3GNT5* #1: F, GAUCAAAGGUACAAUGAUA and R, UAUCAUUGUACCUUUGAUC; si*B3GNT5* #2: F, CAGACUUGAGUGGAUAUGA and R, UCAUAUCCACUCAAGUCUG. To determine the effects of siRNA delivery, a scrambled siRNA (silencer negative control, SN‐1002; Bioneer), which lacks significant sequence similarity and does not target human gene sequences, was used. For *B3GNT5* knockdown, the GSC11 cells were seeded at a density of 5 × 10^5^ cells/well in 6‐well plates. After 24‐hour incubation, the cells were transfected with 10 nmol/L of specific *B3GNT5* siRNAs or scrambled siRNA according to the procedures recommended for Lipofectamine^®^ RNAiMAX Reagent (Invitrogen).

### 2.5 Tumorsphere assay

GSCs transfected with siRNA as described above were seeded onto 24‐well plates at a density of 250 cells/well, followed by incubation for 10 days in the presence of 5% CO_2_ at 37°C without disturbing the plates and without replenishing the medium. Growth factors (bFGF and EGF) were added once every three days. At the end of 10‐day incubation, tumorspheres were collected at the center of the well by slowly swirling the plates, and the images were obtained to determine the number and size of tumorspheres using a digital microscope (Logos Biosystems, Anyang‐si). The number of tumorspheres measuring more than 50 μm in diameter each was counted.

### 2.6 Statistical analysis

Microsoft Excel 2013 (Microsoft Inc), SPSS 20 (SPSS Inc), and GraphPad Prism 5 software (GraphPad Software Inc) were used for statistical analyses. *P* value < .05 indicated statistical significance. The heat maps were created using Java TreeView (version 1.1.6r4). Significant quantitative differences between and among groups were determined by a two‐tailed *t* test and one‐way ANOVA, respectively, followed by Tukey's multiple comparison test. Kaplan‐Meier survival analysis was used to estimate the survival distribution, followed by the log‐rank test to evaluate the differences between stratified groups, using the median value as the cutoff.

## 3 RESULTS

### 3.1 Expression of B3GNT5 in glioma stem cells was decreased in differentiated tumor cells

**FIGURE 1:**
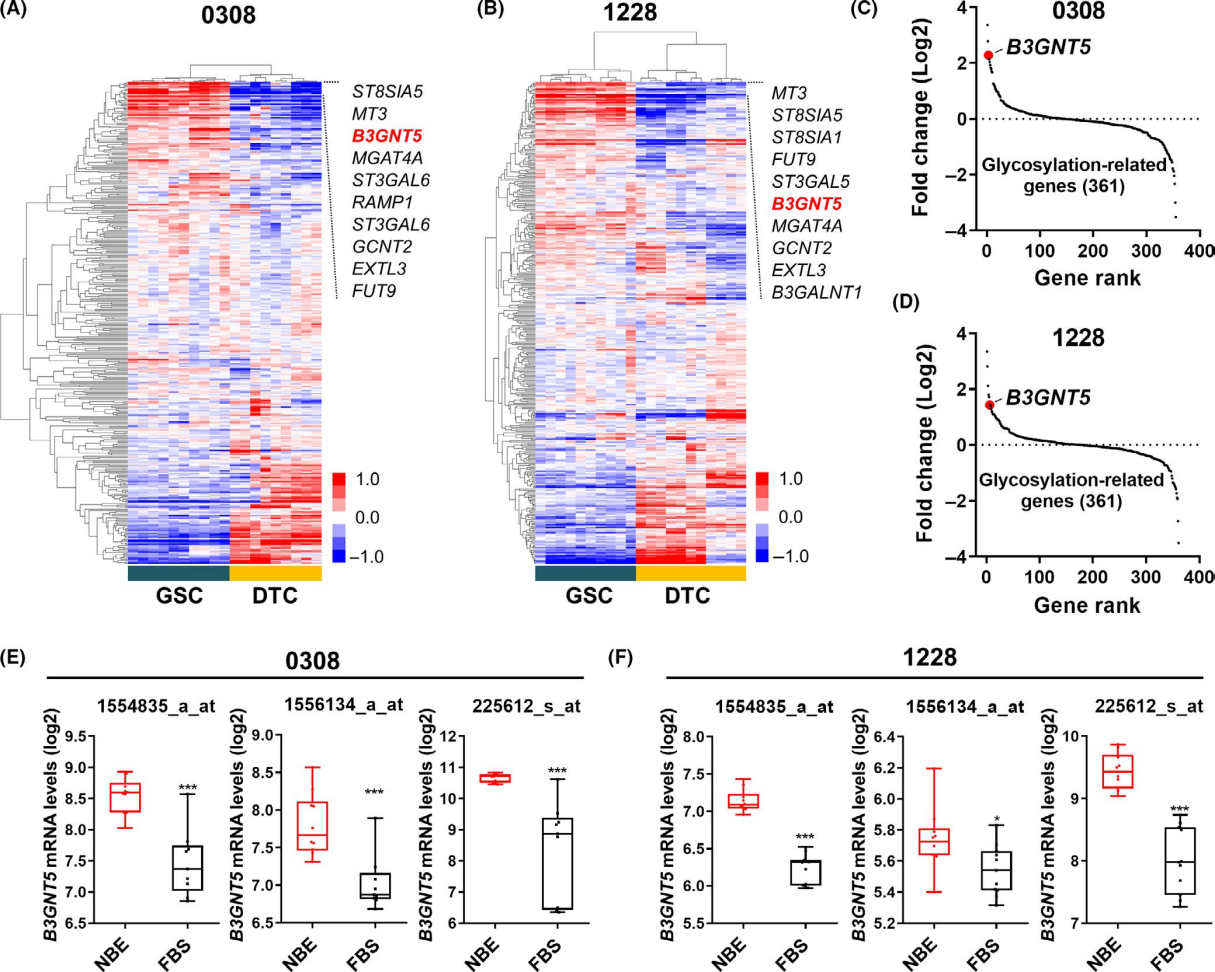
*B3GNT5* was highly expressed in stem cell condition (NBE) among the glycosylation‐related genes. A and B, Heatmap describes the different expression levels of glycosylation‐related genes in GSCs cultured under NBE and FBS conditions according to GSE4536 dataset. A, 0308 cell; B, 1228 cell. C and D, GSE4536 dataset indicated that *B3GNT5* was one of the top‐ranked glycosylation‐related genes in GSCs cultured under NBE and FBS conditions. C, 0308 cell; D, 1228 cell. E and F, The comparison of *B3GNT5 *expression in GSCs cultured under NBE and FBS conditions according to multiple probe sets of GSE4536 dataset. E, 0308 cell; F, 1228 cell. Data are means ± SEM (NBE, n = 10; FBS, n = 11). ^*^ *P* < .05, ^***^ *P* < .001

Using the available dataset, we first analyzed the expression of 361 glycosylation‐related genes between glioma stem cells and their matched differentiated tumor cells. Heatmap and clustering analysis revealed that *ST8SIA5*, *MT3*, *B3GNT5*, and *MGAT4A* were commonly upregulated in both GSC0308 and 1228 in GSCs compared with the differentiated cells (Figure 1A,B). We selected B3GNT5 as one of the top‐ranked genes according to its fold change (Figure 1C,D) and confirmed its significant expression using three different microarray probes (Figure 1E,F). The results suggest that *B3GNT5* exhibited glioma stem cell properties.

### 3.2 Validation of B3GNT5 expression in GSCs

**FIGURE 2:**
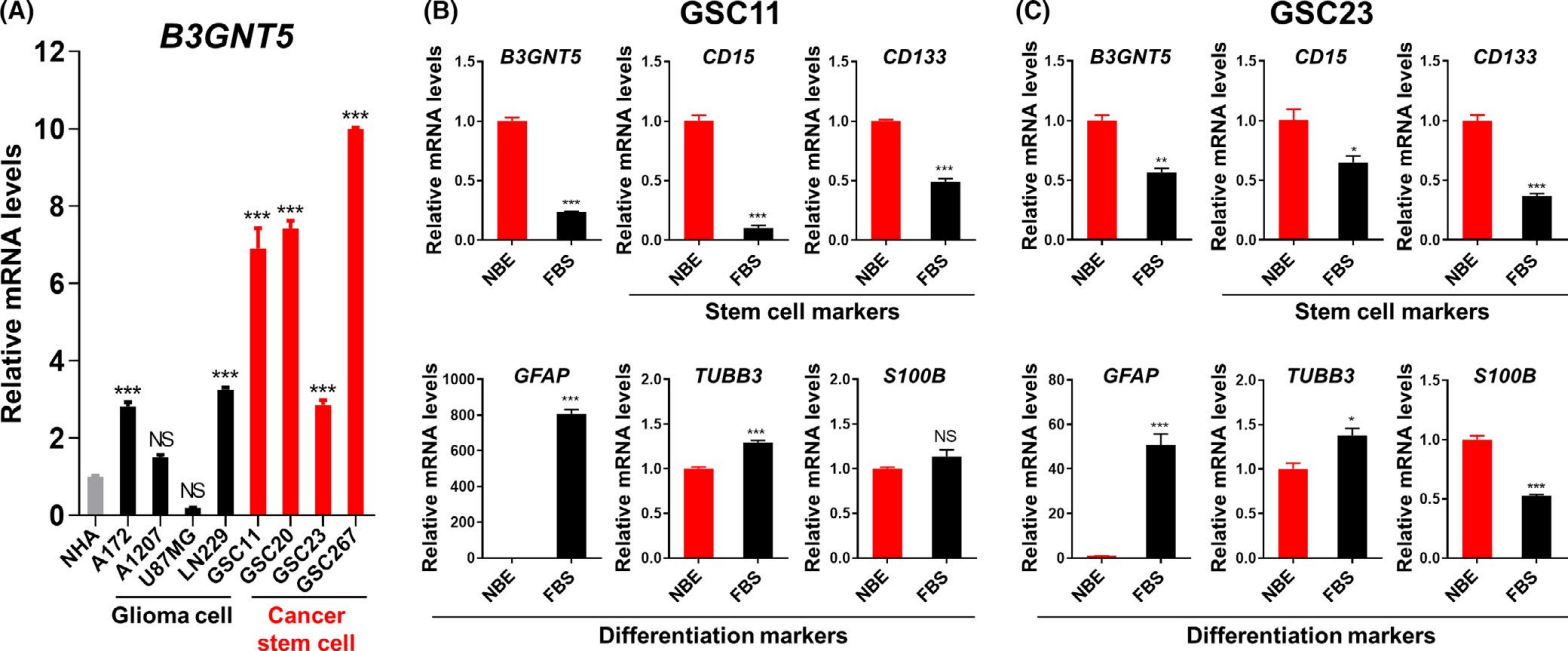
Expression of *B3GNT5* is highly correlated with the stemness of GSCs. A, RT‐qPCR analysis of mRNA expression of *B3GNT5* in normal human astrocyte (NHA), glioma cells, and cancer stem cells. Data are means ± SEM (n = 3). ^*^ *P* < .05, ^***^ *P* < .001. B and C, RT‐qPCR analysis of *B3GNT5*, *CD15*, *CD133*, *GFAP*, *TUBB3*, and *S100B* mRNA expressions in NBE‐ and FBS‐cultured GSCs. Data are means ± SEM (n = 3). ^*^ *P* < .05, ^**^ *P* < .01, ^***^ *P* < .001. B, GSC11; C, GSC23

We compared the levels of *B3GNT5* mRNA expression in normal human astrocytes (NHAs), glioma cell lines (A172, A1207, U87MG, and LN229), and patient‐derived GSCs (GSC11, 20, 23, and 267) (Figure 2A). Real‐time PCR analysis showed that *B3GNT5* was significantly upregulated in GSCs compared with normal astrocytes and glioma cell lines. Using matched cultures of GSCs or their differentiated cells, we confirmed a strong upregulation of B3GNT5 mRNA in GSCs compared with matched differentiated cells and stem cells (Figure 2B,C). GSC markers (ie, CD133, and CD15), astrocytes (GFAP), and neuronal marker (TUBB3) were used as controls. The data demonstrate the dramatic upregulation of *B3GNT5* in GSCs.

### 3.3 Silencing of B3GNT5 inhibits neurosphere formation of GSCs

**FIGURE 3:**
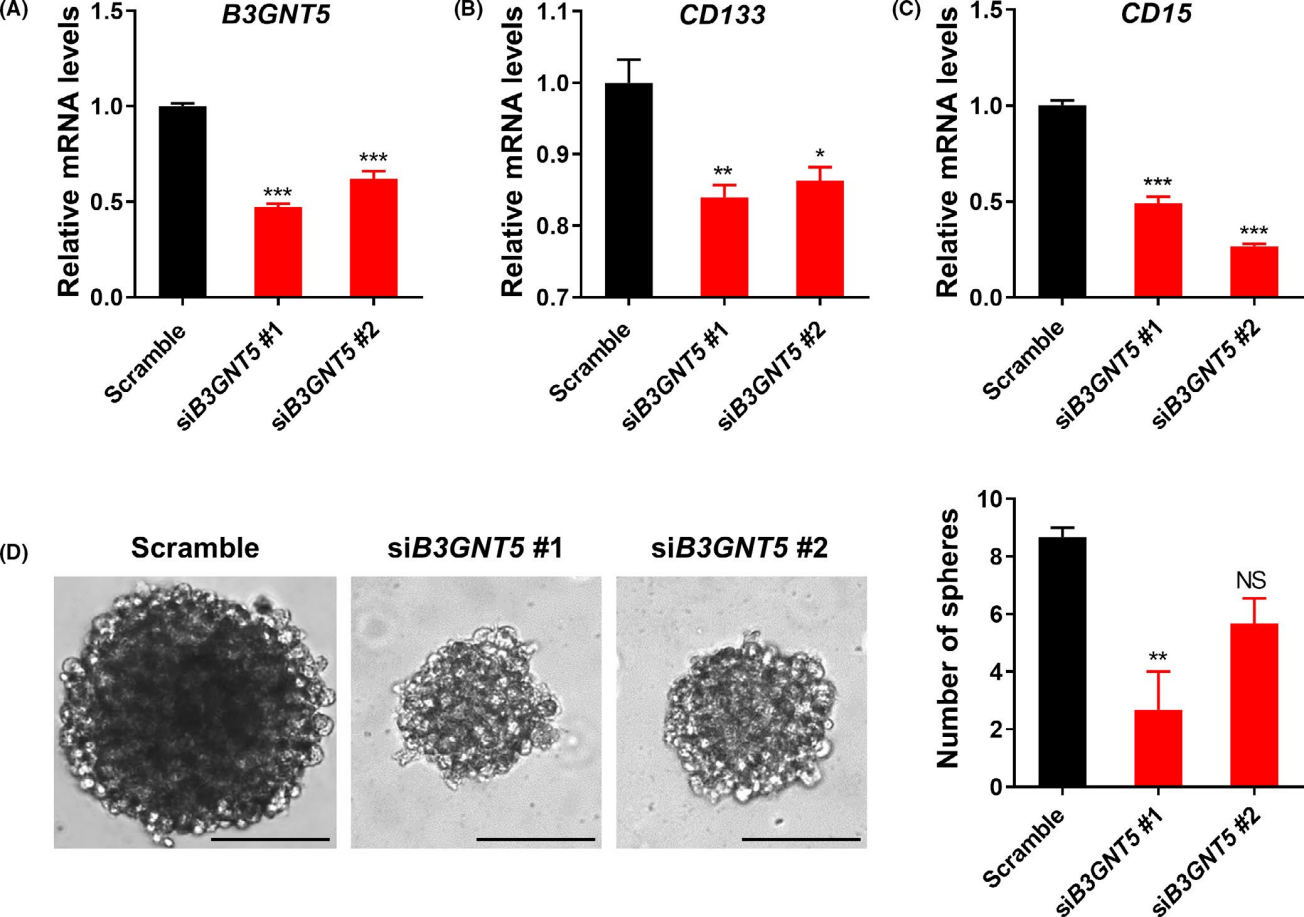
Silencing of *B3GNT5* inhibits the stemness of GSCs. A, RT‐qPCR analysis showing *B3GNT5* knockdown after transfection with siRNA in GSC11. Data are means ± SEM (n = 3). ^***^ *P* < .001. B and C, mRNA expression levels of *CD133* (B) and *CD15* (C) in si*B3GNT5*‐treated GSC11. Data are means ± SEM (n = 3). ^*^ *P* < .05, ^**^ *P* < .01, ^***^ *P* < .001. D, Effects of *B3GNT5* knockdown on neurosphere formation in GSC11. Data are means ± SEM (n = 3). ^**^ *P* < .01. Scale bars represent 100 μm

In order to elucidate the function of *B3GNT5* in the maintenance of stem cell properties of GSCs, we designed two small interfering RNAs (siRNA) to silence the *B3GNT5*. Real‐time PCR analysis showed that si*B3GNT5* #1 and si*B3GNT5* #2 inhibit *B3GNT5* mRNA levels by 38% and 53% in GSC11, respectively (Figure 3A). Knockdown of *B3GNT5* decreased GSC markers (CD133 and CD15) mRNA levels (Figure 3B,C) as well as neurosphere formation (Figure 3D) compared with the scramble control indicating that *B3GNT5* was required for self‐renewal of GSC11.

### 3.4 B3GNT5 is highly expressed in GBM

**FIGURE 4:**
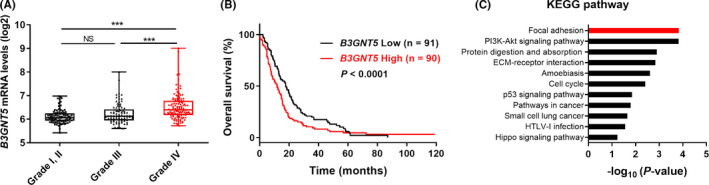
*B3GNT5* is highly expressed in GBM. A, The comparison of *B3GNT5 *expression by GBM grade using REMBRANDT RNAseq dataset. Data are means ± SEM (nontumor, n = 28; astrocytoma, n = 147; oligodendroglioma, n = 67; GBM, n = 219; Grade I and II, n = 100; Grade III, n = 85; Grade IV, n = 130). ^***^ *P* < .001. B, Kaplan‐Meier survival curves of 181 GBM patients (*B3GNT5* high, n = 91; *B3GNT5* low, n = 90, *P* < .0001) in the REMBRANDT microarray dataset. C, Kyoto Encyclopedia of Genes and Genomes (KEGG) pathway analysis for upregulated genes related to high expression of *B3GNT5* in the REMBRANDT microarray dataset

In order to understand the clinical significance of *B3GNT5*, we analyzed *B3GNT5* mRNA levels based on histology and grade in a Repository for Molecular Brain Neoplasia Data (REMBRANDT) database. The expression of *B3GNT5* was the highest in GBM compared with low‐ and high‐grade gliomas (Figure 4A). More importantly, higher levels of B3GNT5 were associated with significantly shorter times (Figure 4B, *P* < .0001). These results indicate that B3GNT5 is a potentially useful marker for evaluation of GBM patient survival.

To elucidate the related signaling pathways of *B3GNT5*, we listed up to 45 upregulated genes showing elevated *B3GNT5* expression in the REMBRANDT dataset. Kyoto Encyclopedia of Genes and Genomes (KEGG) pathway analysis results showed that upregulated differentially expressed genes were significantly enriched in focal adhesion, PI3K‐Akt signaling pathway, and ECM‐receptor interaction (Figure 4C).

### 3.5 B3GNT5 is overexpressed in classical and mesenchymal subtypes

**FIGURE 5:**
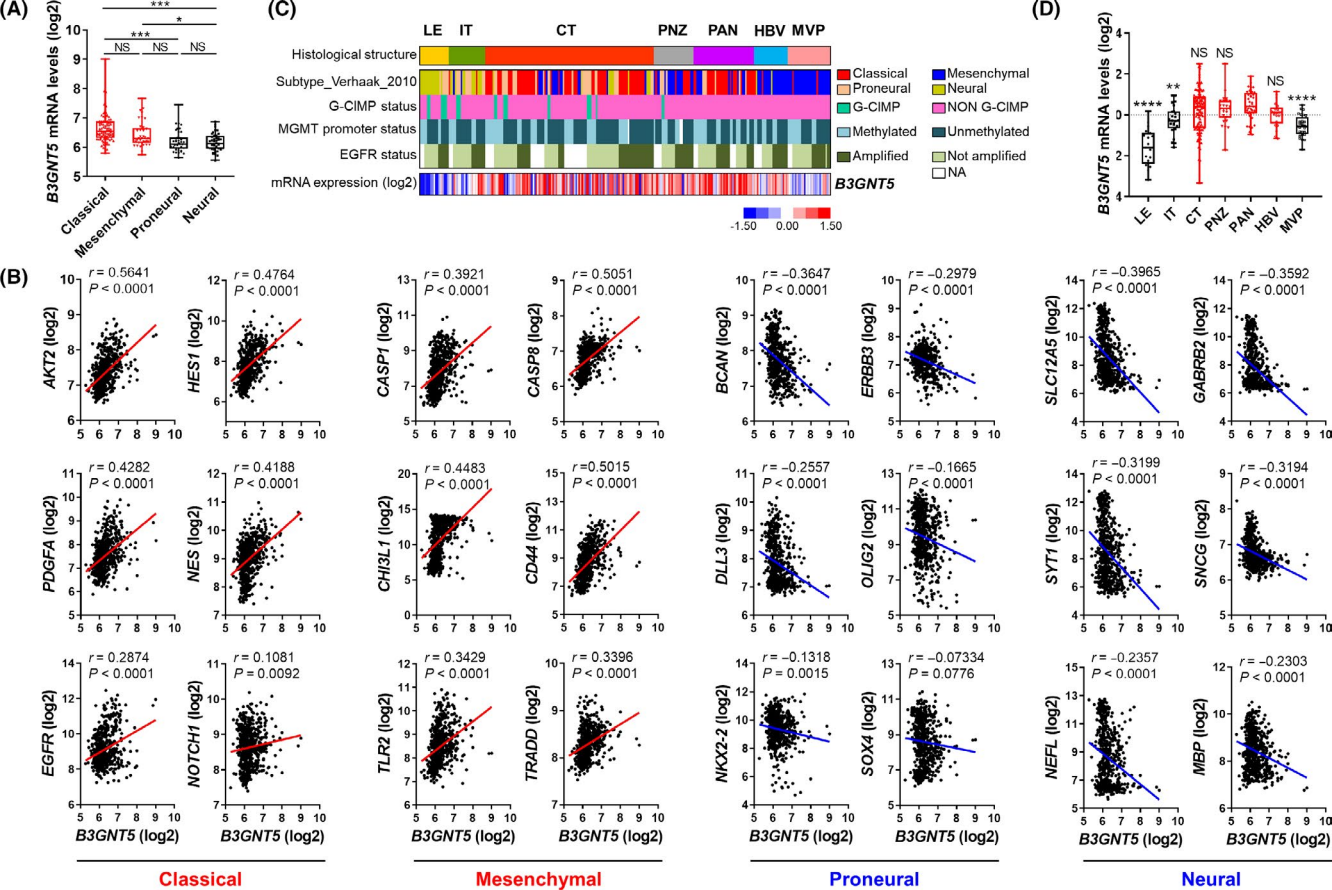
*B3GNT5* is overexpressed in classical and mesenchymal subtypes. A, Comparison of *B3GNT5* mRNA levels among the groups with GBM subtypes (Classical, n = 99; Mesenchymal, n = 37, Neural, n = 39; Proneural, n = 44). ^*^ *P* < .05, ^***^ *P* < .001. B, The correlation analysis between *B3GNT5* expression was performed with classical, mesenchymal, proneural, and neural GSC markers in REMBRANDT RNAseq dataset. C, Heatmap of *B3GNT5* expression signature correlated with GBM region in the Ivy GAP RNAseq dataset. LE, leading edge; IT, infiltration tumor; CT, cellular tumor; PNZ, perinecrotic zone; PAN, pseudopalisading cells around necrosis; HBV, hyperplastic blood vessels; MVP, microvascular proliferation. D, *B3GNT5* mRNA expression by different regions of GBM in the Ivy GAP RNAseq dataset. Data are means ± SEM (LE, n = 19; IT, n = 24; CT, n = 111; PNZ, n = 26; PAN, n = 40; HBV, n = 22; MVP, n = 28). LE, leading edge; IT, infiltration tumor; CT, cellular tumor; PNZ, perinecrotic zone; PAN, pseudopalisading cells around necrosis; HBV, hyperplastic blood vessels; MVP, microvascular proliferation. ^**^ *P* < .01 relative to the PAN group, ^****^ *P* < .0001 relative to the PAN group

There are four different GBM subtypes—proneural, classical, mesenchymal, and neural—which are defined on the basis of genome‐wide analysis of mRNA expression in more than 300 GBM patient tissues.^16^ These subtypes also exhibit their characteristic tumor histology and molecular alterations, and are associated with unique clinical outcomes. Using the REMBRANDT database, we investigated whether B3GNT5 was enriched in specific patient cohorts. As shown in Figure 5A, the *B3GNT5* mRNA was enriched in classical and mesenchymal subtypes. Consistently, the public RNAseq data revealed (Figure 5B) that *B3GNT5* mRNA levels were positively correlated with classical (*AKT2*, *HES1*, *PDGFA*, *NES*, *EGFR*, and *NOTCH1*) and mesenchymal (*CASP1*, *CASP8*, *CHI3L1*, *CD44*, *TLR2*, and *TRADD*) GBM markers, but negatively correlated with proneural (*BCAN*, *ERBB3*, *DLL3*, *OLIG2*, *NKX2‐2*, and *SOX4*) and neural GBM markers (*SLC12A5*, *GABRB2*, *STY1*, *SNCG*, *NEFL*, and *MBP*). In order to understand the histopathological significance of *B3GNT5* expression, we investigated *B3GNT5* expression in GBM in terms of regional differences using the Ivy Glioblastoma Atlas Project dataset (Figure 5C,D). The dataset includes mRNA expression profiles collected from the following tumor areas: leading edge (LE), infiltrating tumor (IT), cellular tumor (CT), perinecrotic zone (PNZ), pseudopalisading cells around necrosis (PAN), hyperplastic blood vessels (HBV), and microvascular proliferation (MVP). Interestingly, the expression of *B3GNT5* was significantly upregulated in CT, PNZ, and PAN, but downregulated in LE, IT, and MVP. The highest expression was found in PAN, which is associated with hypoxia, cell migration, and immune response.^17^

In conclusion, we demonstrated that elevated *B3GNT5* expression was associated with GBM aggressiveness and maintenance of self‐renewal in glioma stem cells. We propose that *B3GNT5* is a marker of poor prognosis in GBM.

## 4 DISCUSSION

GBM is one of the most aggressive tumors and is characterized by cellular heterogeneity, tumor infiltration into normal tissues, necrosis, and angiogenesis. It has been reported that unique tumor cell populations known as CSCs serve as the primary source of tumor initiation and increase the resistance of tumor to chemotherapy and radiotherapy. Recently, treatment with bone morphogenic protein‐4 (BMP‐4)—which is known to promote astrocyte differentiation—suppressed tumorigenicity in the GBM by inducing differentiation of CSCs.^18^ We therefore sought to identify molecular contributors related to CSCs with a high level of expression involving the neural stem or progenitor cells. It has been shown that genetic mutations in normal stem or progenitor cells generate CSCs, which grow and differentiate into primary tumors. Furthermore, similar to normal stem cells, CSCs represent a small CD133‐positive fraction of GBM that contribute to a heterogenous cell population, which proliferates indefinitely.^2^

In this study, we found that *B3GNT5* expression was significantly downregulated during GSC differentiation after serum supplementation. Previously, it was reported that neurobasal media containing EGF and bFGF (NBE) used to culture undifferentiated GSCs exhibit more robust tumorigenic potential, heterogenous morphology, and indefinite potential for self‐renewal compared with serum‐cultured differentiated GSCs.^14^ Lee et al also have shown that FBS‐cultured cells lost their tumorigenic potential in the stereotaxic GBM mouse models.^14^ This finding indicated that B3GNT5 induced tumorigenesis by ensuring self‐renewal of GSCs. *B3GNT5* was highly expressed in human GBM and glioma cell lines in culture, and was associated with poor patient survival. Consistent with these findings, depletion of *B3GNT5* using siRNA in patient GBM‐derived GSCs decreased neurosphere formation and migration activity. The effects of *B3GNT5* knockdown on tumorigenicity require further investigation using nude mice. We showed that *B3GNT5* expression was even more robust in enriched GSCs than in the cell population from which they were originally derived.

Another important finding is that B3GNT5 expression was closely related to focal adhesion and ECM‐receptor interaction based on bioinformatics analysis. Based on these observation, we hypothesized that B3GNT5 may participate in regulating cell invasion to their local microenvironment of GBM. Due to its important role in the progression of glioma, B3GNT5 may serve as a novel candidate molecule for developing new strategies against glioma.

GSLs are found abundantly on cellular membranes and contain either a ceramide or a sphingoid as the hydrophobic lipid moiety. GSL series are functionally important in cell adhesion, cell migration, regulation of signaling proteins, and binding of pathogens and toxins.^4^, ^5^ GSLs are classified into different series according to their core structure. Based on the core structure, GSLs exist as ganglio‐series (Galβ1‐3GalNAcβ1‐4Galβ1‐4Glcβ1‐1′Cer), globo‐ (GalNAcβ1‐3Galα1‐4Galβ1‐4Glcβ1‐1′Cer), isoglobo‐series (GalNAcβ1‐3Galα1‐3Galβ1‐4Glcβ1‐1′Cer), muco‐series (Galβ1‐4Galβ1‐4Glcβ1‐1′Cer), and lacto‐series (Galβ1‐3GlcNAcβ1‐3Galβ1‐4Glcβ1‐1′Cer). Among the GSL series, β1‐3‐linked GlcNAc structure exists only in the core structure of lacto‐series GSL. Since our study demonstrated the upregulation of B3GNT5 in GSCs, an abundance of lacto‐ or neolacto‐based GSL structures exist on GSCs.

Sulfoglucuronyl glycolipids (SGGL) such as SGGL‐1 and SGGL‐2 (SGGL‐1; SO_4_3‐GlcAβ1‐3Galβ1‐4GlcNAcβ1‐3Galβ1‐4Glcβ1‐1Cer, SGGL‐2; SO_4_3‐GlcAβ1‐3Galβ1‐4GlcNAcβ1‐3Galβ1‐4GlcNAcβ1‐3Galβ1‐4Glcβ1‐1Cer) represent extended structures of B3GNT5‐mediated nLc GSL. SGGL are detected during brain development and reported to play a critical role in cell‐cell interaction in central nervous system.^19^ B3GNT5 is the key enzyme involved in initiating the formation of root structure of the Lewis X carbohydrate Galβ1‐4 (Fucα1‐3)GlcNAcβ1‐R, which mediates cellular adhesion and differentiation of the neuronal system.^20^ Since our study demonstrated the elevated expression of B3GNT5 in GSCs, a comparative structural analysis of B3GNT5‐based glycolipids such as SGGL and Lewis X on GSCs will be performed in our next study to elucidate the structure of the major glycolipid controlling the stemness of glioma stem cells.

In conclusion, our results indicate the potential of B3GNT5 as a prognostic biomarker used to characterize the most aggressive cells in GBM, with a possible therapeutic role in the targeted therapy of GBM.

## CONFLICT OF INTEREST

The authors have no conflict of interest to declare.
